# Changes in inpatient payer-mix and hospitalizations following Medicaid expansion: Evidence from all-capture hospital discharge data

**DOI:** 10.1371/journal.pone.0183616

**Published:** 2017-09-28

**Authors:** Seth Freedman, Sayeh Nikpay, Aaron Carroll, Kosali Simon

**Affiliations:** 1 School of Public and Environmental Affairs, Indiana University, Bloomington, Indiana, United States of America; 2 Department of Health Policy, Vanderbilt University Medical Center, Nashville, Tennessee, United States of America; 3 Department of Pediatrics, School of Medicine, Indiana University, Indianapolis, Indiana, United States; University of Rhode Island, UNITED STATES

## Abstract

**Context:**

The Affordable Care Act resulted in unprecedented reductions in the uninsured population through subsidized private insurance and an expansion of Medicaid. Early estimates from the beginning of 2014 showed that the Medicaid expansion decreased uninsured discharges and increased Medicaid discharges with no change in total discharges.

**Objective:**

To provide new estimates of the effect of the ACA on discharges for specific conditions.

**Design, setting, and participants:**

We compared outcomes between states that did and did not expand Medicaid using state-level all-capture discharge data from 2009–2014 for 42 states from the Healthcare Costs and Utilization Project’s FastStats database; for a subset of states we used data through 2015. We stratified the analysis by baseline uninsured rates and used difference-in-differences and synthetic control methods to select comparison states with similar baseline characteristics that did not expand Medicaid.

**Main outcome:**

Our main outcomes were total and condition-specific hospital discharges per 1,000 population and the share of total discharges by payer. Conditions reported separately in FastStats included maternal, surgical, mental health, injury, and diabetes.

**Results:**

The share of uninsured discharges fell in Medicaid expansion states with below (-4.39 percentage points (p.p.), -6.04 –-2.73) or above (-7.66 p.p., -9.07 –-6.24) median baseline uninsured rates. The share of Medicaid discharges increased in both small (6.42 p.p. 4.22–6.62) and large (10.5 p.p., 8.48–12.5) expansion states. Total and most condition-specific discharges per 1,000 residents did not change in Medicaid expansion states with high or low baseline uninsured rates relative to non-expansion states (0.418, p = 0.225), with one exception: diabetes. Discharges for that condition per 1,000 fell in states with high baseline uninsured rates relative to non-expansion states (-0.038 95% p = 0.027).

**Conclusions:**

Early changes in payer mix identified in the first two quarters of 2014 continued through the Medicaid expansion’s first year and are distributed across all condition types studied. We found no change in total discharges between Medicaid expansion and non-expansion states, however residents of states that should have been most affected by the Medicaid expansion were less likely to be hospitalized for diabetes.

## Introduction

One of the main ways that the Affordable Care Act (ACA) sought to decrease the uninsured rate was to increase Medicaid coverage through an expansion of the program to low-income, primarily childless adults. Even though a significant number of states chose not to participate in the expansion, 9.1 million newly eligible adults have obtained coverage through this mechanism [1]. Many have wondered how this expansion might change the way hospitals are paid for inpatient admissions. Increased access to care might increase admissions, as more people can pay for necessary care. However, increased access to outpatient care might also lessen the need for inpatient admissions. Additionally, the impact of the expansion on different conditions may vary because of wide adoption of care models that foster coordination between hospitals and other providers. As more patients with chronic or complex health problems become eligible for coverage, patterns of hospitalization could change [2].

It is also possible that the expansion of Medicaid could affect how hospitals are paid for inpatient care. It is therefore important to determine how potential increases or decreases for Medicaid admissions come out of the share of private insurance or uninsured admissions.

The objective of this study was to determine how coverage for inpatient hospital admissions changed with the Medicaid expansion (both as a share of total hospitalizations as well as the absolute number of hospitalizations), to examine how this change differed by extent of expansion itself, and to see how these changes varied by disease.

## Methods

The FastStats data comprise state-by-quarter counts of total hospital discharges for community-residing patients from 42 all-capture state databases for the period 2009–2014 (N = 1,008) [3]. All states were included in the analysis because they reported data through the 4^th^ quarter of 2014. Our measures include total discharges (per 1,000 population) and the share of total non-Medicare hospital discharges by payer (private insurance, Medicaid, or uninsured). FastStats also provides counts of discharges for five subcategories: medical, surgical, maternal, injury, and mental health/substance abuse. FastStats reports data for three subcategories of medical discharges: diabetes, congestive heart failure, and asthma, though congestive heart failure and asthma data were missing for too many states to include in our analysis (see S2 Table). The ICD-9 codes used to make these determinations have been used in previous work and are available in the S1 Table.

We used a difference-in-differences design to compare the change in discharges in Medicaid expansion states on and after the first quarter of 2014 to the change in non-expansion states. Our sample included 22 expansion states (AR, AZ, CA, CO, HI, IA, IL, KY, MA, MD, MI, MN, ND, NJ, NM, NV, NY, OR, RI, VT, WA, WV) and 20 non-expansion states (FL, GA, IN, KS, LA, ME, MO, MT, NC, NE, OK, PA, SC, SD, TN, TX, UT, VA, WI, WY). Note that we categorized Michigan as treated for all of 2014, even though it expanded in the second quarter of 2014. We obtained information on state expansion status and timing from the Kaiser Family Foundation [4]. The post-expansion period was defined as Q1 of 2014 through Q4 of 2014. We omitted Q4 of 2013 because of the welcome-mat effect: people who were previously eligible for Medicaid began to enroll before the ACA’s major expansion took place [5]. All analyses used multivariable ordinary least squares regression with quarter-by-year indicators to account for common time trends and seasonality and state indicators to account for differences across states. Our results are robust to controlling for the age, sex, marital status, income and education distributions of the state as well as the unemployment rate (S3 Fig). All regressions used state weights based on 2014 population, and standard errors were clustered at the state level. To account for the small number of clusters in our analysis, we estimated p-values that account for few-cluster bias using a wild-cluster bootstrap [6]. We found that the results were robust to alternate p-values (results are provided in the S4 Table).

We hypothesized that the change in discharges was proportional to the change in the uninsured population after expansion. Therefore, we divided the expansion states into those with 2013 uninsured rates below that year’s median (HI, IA, IL, KY, MA, MD, MI, MN, NY, RI, VT, WA, WV) as well as above it (AR, AZ, CA, CO, ND, NJ, NM, NV, OR) and conducted our analyses separately for these two groups of expansion states. We called the former group of states “small expansion” states and the latter group “large expansion states”.

A necessary assumption of difference-in-differences is that the dependent variables were trending similarly between Medicaid expansion and non-expansion states before 2014. We tested this assumption (S4 Fig) and found no evidence of differential pre-trends for all payer share outcome variables except a small differential trend for the uninsured share of surgical discharges in small expansion states. However, we did find evidence of distinct differential pre-trends for some outcomes measuring the number of visits per 1,000 population (S6 Fig). We therefore used synthetic control, a data-driven method of choosing a set of non-expansion states for the control group, for the number of visits outcomes [7]. Briefly, synthetic control selects and applies a set of weights that minimizes the difference in the pre-period values between treatment and control units before the treatment, and we implemented this match using all pre-period quarters of the respective outcome variable. Because this method requires a fully balanced panel, we excluded North Dakota for all visit outcomes and Wyoming for the diabetes outcome due to some quarters of missing data. We used Fisher permutation tests to obtain p-values [8–10]

As a further sensitivity analysis, we analyzed data through Q3 of 2015 for the 17 states that report through this time period. We found the results did not change (S2 Fig and S3 Table).

## Results

**Fig 1:**
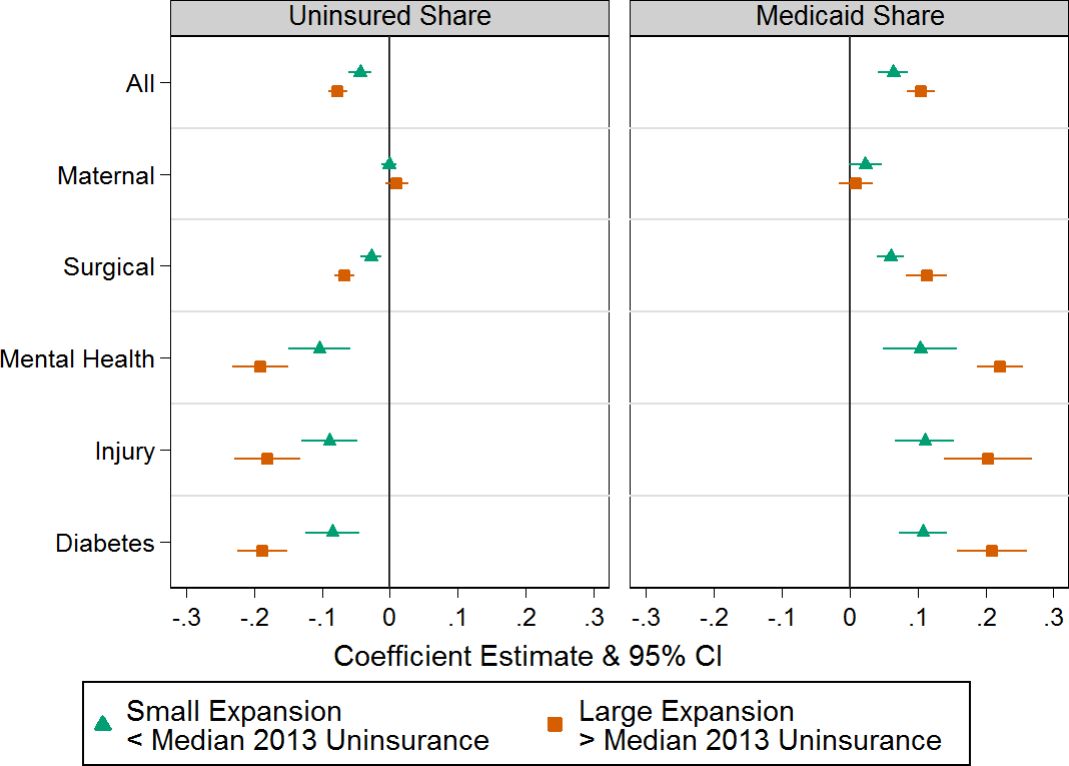
Difference-in-difference estimates of effect of Medicaid expansion on uninsured and Medicaid shares. The figure presents regression-adjusted difference-in-difference estimates and their 95% confidence intervals by discharge type. Information on adjusted regression specification may be found in the S1 Appendix. Small expansion states include HI, IA, IL, KY, MA, MD, MI, MN, NY, RI, VT, WA, WV, large expansion states include AR, AZ, CA, CO, ND, NJ, NM, NV, OR, and nonexpansion states include FL, GA, IN, KS, LA, ME, MO, MT, NC, NE, OK, PA, SC, SD, TN, TX, UT, VA, WI, WY. Payer mix is the share of non-Medicare hospital discharges covered by Medicaid and with no source of coverage. Standard errors are heteroscedasticity robust and clustered at the state level. Results are weighted by 2014 state population (N = 1008).

Fig 1 displays difference-in-difference estimates of the impact of the Medicaid expansion on hospital payer mix. The uninsured share of total discharges decreased relative to non-expansion states in both small expansion states (-4.39 p.p., -6.04 –-2.73) and large expansion states (-7.66 p.p., -9.07 –-6.24). The Medicaid share of total discharges increased relative to non-expansion states in both small (6.42 p.p., 4.22–6.62) and large expansion states (10.5 p.p., 8.48–12.5). For example, in Michigan, a representative small expansion state, the share of uninsured discharges decreased by 3.8 percentage points and the share of Medicaid discharges increased by 6.7 from 2013 to 2014, whereas in Colorado, a representative large expansion state uninsured discharges decreased by 9.0 percentage points and Medicaid discharges increased by 11.3 percentage points. We also found that the private share of total discharges fell slightly in expansion states relative to non-expansion states in both types of states (small: -2.03 p.p., -3.78–-0.28; large: -2.85 p.p., -4.35–-1.34) (S1 Fig).

Among discharge sub-types, there was little change in the uninsured (small: 95% CI: -1.24–0.97; large: 95% CI -0.70–2.67) or Medicaid share (small: 95% CI -0.04–4.69; large: 95% CI -1.63–3.42) of maternal discharges as might have been expected, since pregnancy- related Medicaid eligibility was unaffected by the ACA; we did see shifts in payer mix for other types. Changes in payer mix were generally smaller for surgical discharges than for medical conditions, such as mental health or diabetes. Payer mix also changed significantly for injury-related discharges. In general, changes in payer mix relative to non-expansion states were larger in large expansion states than in small expansion states, further confirming that these effects are likely to be due to expansion rather than contemporaneous or unobserved confounding factors.

**Fig 2:**
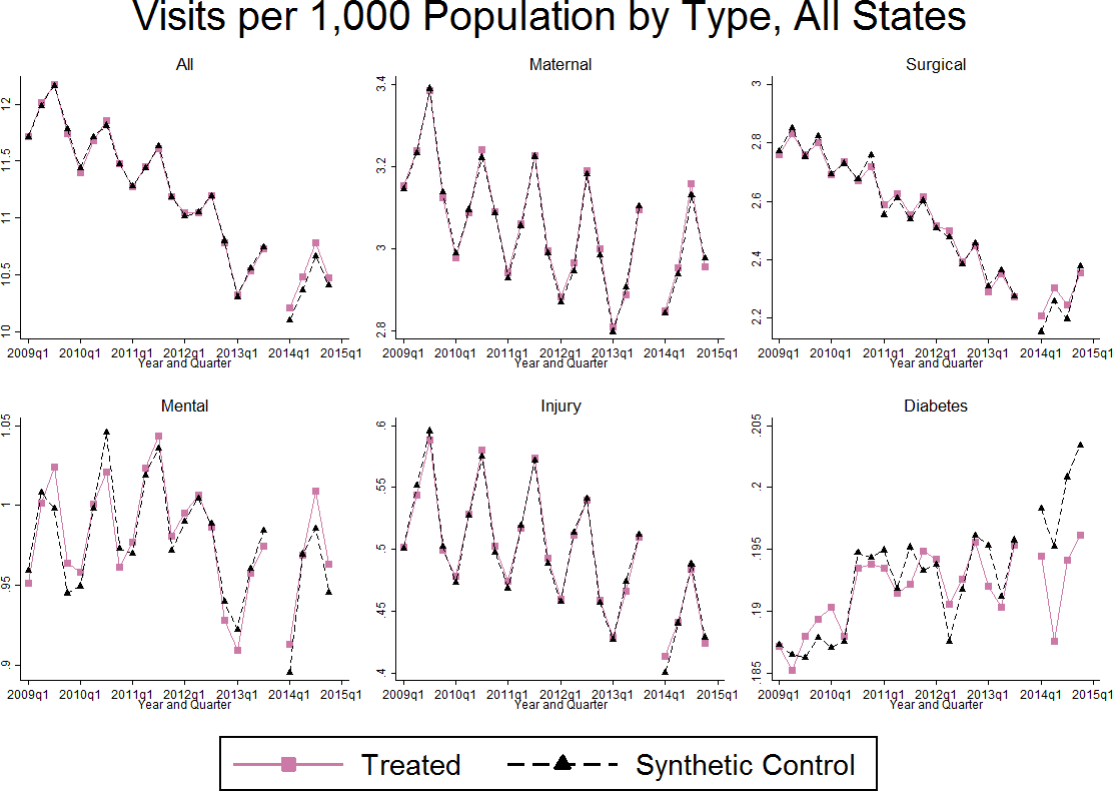
Trends in visits for expansion states vs. synthetic control, all states. The figure presents mean time trends for expansion states weighted by state population in 2014 (treated) and a weighted average of non-expansion states (synthetic controls). The method of choosing weights for the control states is found in the S1 Appendix. Outcomes are based on the number of non-Medicare hospital discharges within each type of visit (N = 1008).

Because we found evidence of differential trends in discharges per 1,000 population for some outcomes, we chose to use synthetic control to pick a subset of non-expansion states as a control group using a data-driven procedure. Fig 2 displays the results. The tight match between treatment and control pre-trends speaks to the performance of synthetic control: the weighted non-expansion states match the control trend almost exactly for each type of discharge. Therefore, the effect of Medicaid expansion can be inferred by the size of the gap between treatment and control groups starting in Q1 of 2014. Table 1 reports estimates for the average difference in treatment and control groups in 2014. For total discharges per 1,000 residents, the gap between treatment and control groups is small and not significantly different than zero (p = 0.268). Total discharges do not change for maternal health (p = 0.593), surgical (p = 0.413), mental health (p = 0.863), or injury-related discharges (p = 0.652) on average. However, there was a striking reduction in diabetes-related discharge in Medicaid expansion states starting in Q1 of 2014, although this reduction is not significant for expansion states on average (p = 0.188) because the difference becomes smaller in later quarters.

**Table 1:** Synthetic control estimates: Effect of Medicaid expansion on visits per 1,000 population.

	All Expansion States		Expansion States with High Uninsured Rates		Expansion States with Low Uninsured Rates	
	Estimate	P-value	Estimate	P-value	Estimate	P-value
All	0.405	[0.268]	0.660	[0.134]	0.131	[0.881]
Maternal	0.026	[0.593]	-0.036	[0.298]	0.049	[0.413]
Surgical	0.127	[0.413]	0.051	[0.328]	0.232	[0.297]
Mental	0.058	[0.863]	-0.022	[0.588]	0.087	[0.955]
Injury	0.005	[0.652]	0.013	[0.511]	-0.016	[0.430]
Diabetes	-0.025	[0.188]	-0.039	[0.027]	-0.021	[0.575]

Notes: The table presents the average difference between expansion states weighted by state population in 2014 and a weighted average of non-expansion states after the Medicaid expansion (four quarters of 2014). The method of choosing weights for the control states are found in the S1 Appendix. Outcomes are based on the number of non-Medicare hospital discharges within each type of visit. Small expansion states include HI, IA, IL, KY, MA, MD, MI, MN, NY, RI, VT, WA, WV, large expansion states include AR, AZ, CA, CO, ND, NJ, NM, NV, OR, and non-expansion states include FL, GA, IN, KS, LA, ME, MO, MT, NC, NE, OK, PA, SC, SD, TN, TX, UT, VA, WI, WY. P-values are calculated based on Fisher permutation tests, as described in the S1 Appendix (N = 1008).

Table 1 also shows estimates on changes by size of expansion. Relative to the non-expansion state control group, there were no changes in total discharges for either large expansion states (0.66, p = 0.134) or small expansion states (0.13, p = 0.881), which strengthens the conclusion that expansion was not influential in changing the total number of hospitalizations. Consistent with Fig 2, there were no changes in Table 1 for discharges for any condition except for diabetes, where the effects were significant only among large expansion states; discharges fell by 0.039 per 1,000 residents (p = 0.027) in large expansion states while the estimated change was not different in small expansion states (-0.021, p = 0.575).

## Discussion

These results suggest that early changes in hospital payer mix in 2014 were sustained [11–12]. We also found that payer mix changed in expected ways for surgical, injury, and condition-related discharges and that the impact of the expansion on payer mix differs across baseline uninsured rates. Although we found no change in total discharges, we did find a reduction in diabetes-related discharges.

This result is surprising because individuals with diabetes were more likely to have insurance than those without diabetes before the ACA [13]. However, diabetes has increased significantly among low-income populations over the last several decades [14], and today half of all uninsured adults with diabetes have incomes low enough to qualify for Medicaid under the ACA [15]. Indeed, recent work demonstrates that adults were more likely to have been diagnosed with diabetes in Medicaid expansion states after the Medicaid expansion and that prescriptions for diabetes management increased the most, of all medication categories, under Medicaid after 2014 in expansion states [16–17]. If the Medicaid expansion resulted in better access to care for undiagnosed patients with diabetes, then it is possible that hospital utilization could change as well. Many of those newly eligible for Medicaid under the ACA were offered Medicaid managed care, which may better coordinate care of beneficiaries and thus result in avoided hospitalizations.

To gauge the impact of the Medicaid expansion on costs, we conducted a back-of-the-envelope calculation using data from the HealthCare Cost and Utilization Project data. In 2008 the average cost of an inpatient stay where diabetes was listed as the primary or secondary diagnosis was $10,937, or $12,581 when adjusted to 2017 dollars. We found that diabetes discharges fell by 0.038 discharges per 1,000 population in states with high baseline uninsured rates (AR, AZ, CA, CO, ND, NJ, NM, NV, OR). Therefore, a rough estimate of the possible reduction in the cost of inpatient diabetes care is $478 per 1,000 population (-0.038*12581). In Arizona, with a 2016 population of 6,931,071, the estimates imply a potential statewide reduction of $3.3M ($478*6,931,071/1000).

There are several important caveats. First, the estimates of the cost of diabetes care were averaged over all patients. Our results suggest that the reduction in diabetes-related visits was driven by the uninsured who gained Medicaid coverage. However, the cost of diabetes care for the uninsured relative to the average depends on whether uninsured diabetics are healthier than insured diabetics. Second, our estimates use publicly-available data on charges for inpatient care, deflated by a cost-to-charge ratio to infer costs. Therefore they do not represent actual hospital costs.

This study, like all studies, has limitations that warrant consideration. These data are all observational, and, although we have taken efforts to control for other factors, it is possible that some factor other than the Medicaid expansion is the cause of any changes we see. These data are also not fully nationally comprehensive, although they have been used before to make inferences about the United States health care system. We are unable to examine readmission patterns or the use of ambulatory care in between hospitalizations.

Understanding how the ACA has affected total hospitalizations, by specific type and the payer composition facing hospitals, is especially important as policy makers consider changes to the law. Timely availability of hospitalization data covering a large segment of the nation shows that substantial changes in payer composition have occurred, but fewer changes are seen in the number of total hospitalizations, even when comparing large and small expansion experiences. Future research is needed to examine to what extent this reflects increased access among those earlier unable to afford hospital care vs. reduced hospitalizations among those who can substitute towards non-hospital care.

## Supporting information

S1 Table FastStats discharge subcategories. (PDF)

S2 Table Summary of missing data. (PDF)

S3 Table Synthetic control visits per 1,000 population results through Q3 2015. (PDF)

S4 Table Wild cluster bootstrap results. (PDF)

S1 Fig Difference-in-difference estimates of effect of Medicaid expansion on private share. (PDF)

S2 Fig Difference-in-differences payer share results through Q3 of 2015. (PDF)

S3 Fig Difference-in-difference estimates of effect of Medicaid expansion on payer mix with controls. (PDF)

S4 Fig Payer mix pre-trends, no controls. (PDF)

S5 Fig Payer mix pre-trends, with controls. (PDF)

S6 Fig Visits pre-trends, no controls. (PDF)

S7 Fig Additional synthetic control time series. (PDF)

S1 Appendix Empirical methods. (PDF)
