# Supplementary material for: Changes in inpatient payer-mix and hospitalizations following Medicaid expansion: Evidence from all-capture hospital discharge data
Source: PLoS One. 2017 Sep 28;12(9):e0183616. doi: 10.1371/journal.pone.0183616 (PMC5619726; doi:10.1371/journal.pone.0183616)
Supplement: S1 Table — (PDF) [file pone.0183616.s001.pdf]

S1 Table. FastStats Discharge Subcategories

Condition	CCS	DRG Grouper	ICD9
Diabetes	49, 50		
Congestive Heart Failure	108		
Asthma	128		
Maternal	176-196		
Mental health/substance use	650-663, 670		
Injury	660, 661, 662		800-909.2, 909.4, 909.9, 910-994.9, 995.5-995.59, 995.80-995.85, 980.0, 965.00, 965.01, 965.02
Surgical		Surgical	
Medical		Medical and ungroupable DRGs	

Note: All analyses are restricted to community-residing patients and counts are rounded to 50 for conditions with fewer than 50 discharges. The Fast Stats data were adjusted by the Agency for Healthcare Research and Quality for missing data using information from several other datasets including American Hospital Association data, the Trauma Information Exchange Program database, and the American Trauma Society. Discharges are for patients aged 19-64 for uninsured, Medicaid, and privately insured patients and patients aged 65 and older for Medicare. All condition-specific definitions are based on the primary listed diagnosis.
