# Supplementary material for: Changes in inpatient payer-mix and hospitalizations following Medicaid expansion: Evidence from all-capture hospital discharge data
Source: PLoS One. 2017 Sep 28;12(9):e0183616. doi: 10.1371/journal.pone.0183616 (PMC5619726; doi:10.1371/journal.pone.0183616)
Supplement: S2 Table — (PDF) [file pone.0183616.s002.pdf]

S2 Table. Summary of Missing Data

Visit Type	Outcome	State(Number of Missing Quarters)
All	Number of Visits	None
All	Share Medicaid	None
All	Share Uninsured	None
All	Share Private	None
Maternal	Number of Visits	None
Maternal	Share Medicaid	None
Maternal	Share Uninsured	SD(7) VT(23)
Maternal	Share Private	None
Surgical	Number of Visits	None
Surgical	Share Medicaid	None
Surgical	Share Uninsured	VT(4)
Surgical	Share Private	None
Mental Health	Number of Visits	None
Mental Health	Share Medicaid	None
Mental Health	Share Uninsured	VT(4)
Mental Health	Share Private	None
Injury	Number of Visits	None
Injury	Share Medicaid	WY(8)
Injury	Share Uninsured	SD(2) VT(9)
Injury	Share Private	None
Diabetes	Number of Visits	WY(2)
Diabetes	Share Medicaid	MT(3) SD(4) WY(24)
Diabetes	Share Uninsured	HI(24) ME(2) NE(2) RI(4) SD(24) VT(24) WV(3) WY(6)
Diabetes	Share Private	VT(8) WY(14)
CHF	Number of Visits	ME(1) MT(9) SD(3) VT(24) WY(24)
CHF	Share Medicaid	ME(8) MT(22) NE(9) RI(1) SD(23) VT(24) WY(24)
CHF	Share Uninsured	HI(23) IA(21) MA(3) ME(24) MN(22) MT(24) NE(24) NM(3) OR(3) RI(24) SD(24) UT(19) VT(24) WV(21) WY(24)
CHF	Share Private	ME(1) MT(11) RI(1) SD(3) VT(24) WY(24)
Asthma	Number of Visits	ME(4) MT(13) SD(6) VT(21) WY(19)
Asthma	Share Medicaid	KS(1) ME(8) MT(24) NE(16) NM(1) SD(24) UT(4) VT(23) WY(24)
Asthma	Share Uninsured	AR(2) CO(1) HI(24) IA(18) KS(1) KY(2) MA(3) ME(24) MN(17) MT(23) NE(23) NM(4) OR(4) RI(11) SD(24) UT(9) VT(24) WA(2) WV(4) WY(24)
Asthma	Share Private	ME(6) MT(14) RI(1) SD(6) VT(22) WY(19)

Notes: This table reports the number of missing state-quarters by outcome, in addition to ND, which is missing all data for years 2009 and 2010. Given missing data from many state-quarters, we do not report data on CHF or Asthma. For Difference-in-difference analysis we use data from all state-quarters in which data are available. For each outcome analyzed by synthetic controls, we exclude states with any missing data for that outcome.
