# Supplementary material for: Changes in inpatient payer-mix and hospitalizations following Medicaid expansion: Evidence from all-capture hospital discharge data
Source: PLoS One. 2017 Sep 28;12(9):e0183616. doi: 10.1371/journal.pone.0183616 (PMC5619726; doi:10.1371/journal.pone.0183616)
Supplement: S3 Table — (PDF) [file pone.0183616.s003.pdf]

S3 Table. Synthetic Control Visits per 1,000 Population Results through Q3 2015.

	All Expansion States		Expansion States with High Uninsured Rates		Expansion States with Low Uninsured Rates	
	Estimate	P-value	Estimate	P-value	Estimate	P-value
All	0.384	[0.887]	0.162	[0.872]	0.455	[0.854]
Maternal	0.112	[0.404]	0.029	[0.533]	0.231	[0.170]
Surgical	0.221	[0.426]	0.022	[0.936]	0.531	[0.197]
Mental	-0.385	[0.153]	-0.160	[0.133]	-0.581	[0.346]
Injury	0.027	[0.698]	0.035	[0.618]	0.006	[0.799]
Diabetes	-0.035	[0.074]	-0.038	[0.106]	-0.039	[0.094]

Notes: This sample includes the 17 states with data available through the first 3 quarters of 2015. The table presents the average difference between expansion states weighted by state population in 2014 and a weighted average of non-expansion states after the Medicaid expansion (four quarters of 2014 and first three quarters of 2015). The method of choosing weights for the control states are found in the appendix. Outcomes are based on the number of non-Medicare hospital discharges within each type of visit. Small expansion states include HI, IA, KY, MI, MN, NY, large expansion states include CA, CO, NJ, OR, and nonexpansion states include FL, GA, MO, SD, TX, VA, WI. P-values are calculated based on Fisher permutation tests, as described in the appendix.
