# Supplementary material for: Changes in inpatient payer-mix and hospitalizations following Medicaid expansion: Evidence from all-capture hospital discharge data
Source: PLoS One. 2017 Sep 28;12(9):e0183616. doi: 10.1371/journal.pone.0183616 (PMC5619726; doi:10.1371/journal.pone.0183616)
Supplement: S4 Table — (PDF) [file pone.0183616.s004.pdf]

S4 Table. Wild Cluster Bootstrap results

Visit Type	Outcome	Wild Bootstrap P-Value
All	Share Medicaid	<0.001
All	Share Uninsured	<0.001
All	Share Private	0.008
Maternal	Share Medicaid	0.158
Maternal	Share Uninsured	0.599
Maternal	Share Private	0.012
Surgical	Share Medicaid	<0.001
Surgical	Share Uninsured	0.002
Surgical	Share Private	0.002
Mental Health and Substance Abuse	Share Medicaid	0.002
Mental Health and Substance Abuse	Share Uninsured	<0.001
Mental Health and Substance Abuse	Share Private	0.400
Injury	Share Medicaid	<0.001
Injury	Share Uninsured	<0.001
Injury	Share Private	0.006
Diabetes	Share Medicaid	0.002
Diabetes	Share Uninsured	0.002
Diabetes	Share Private	0.024

Notes: The table presents adjusted p-values from a wild-cluster bootstrap t-percentile procedure for the results in Fig 1 and S4 Fig.
