# Supplementary material for: Changes in inpatient payer-mix and hospitalizations following Medicaid expansion: Evidence from all-capture hospital discharge data
Source: PLoS One. 2017 Sep 28;12(9):e0183616. doi: 10.1371/journal.pone.0183616 (PMC5619726; doi:10.1371/journal.pone.0183616)
Supplement: S1 Fig — (PDF) [file pone.0183616.s005.pdf]

S1 Fig. Difference-in-Difference Estimates of Effect of Medicaid Expansion on Private Share.

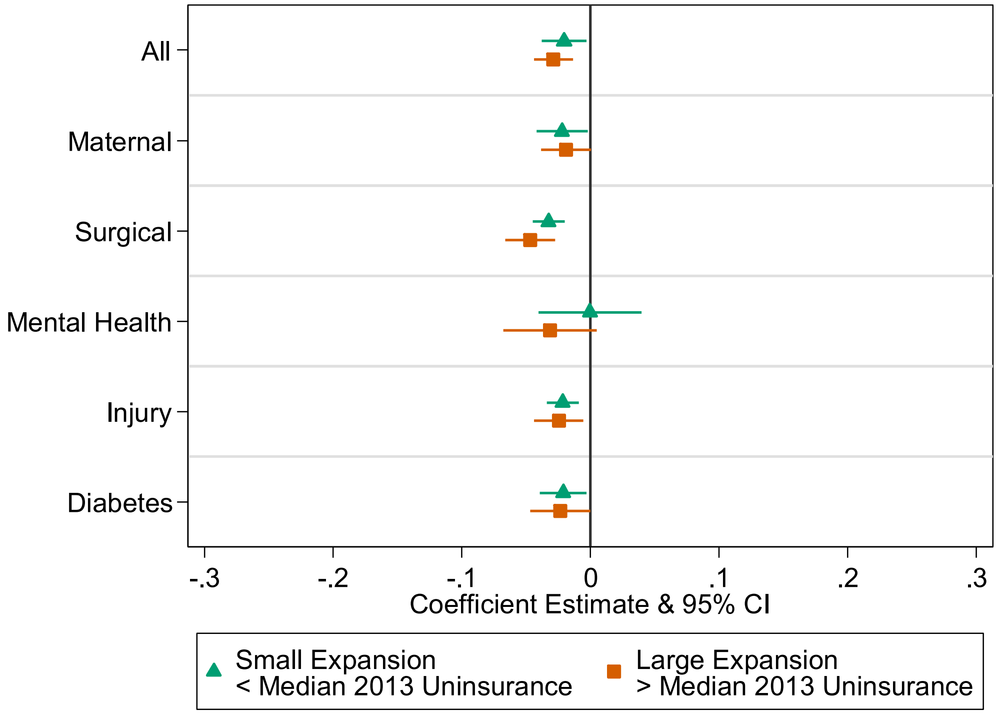

Notes: The figure presents regression-adjusted difference-in-difference estimate and their 95% confidence interval by discharge type. Information on adjusted regression specification may be found in the appendix. Small expansion states include HI, IA, IL, KY, MA, MD, MI, MN, NY, RI, VT, WA, WV, large expansion states include AR, AZ, CA, CO, ND, NJ, NM, NV, OR, and nonexpansion states include FL, GA, IN, KS, LA, ME, MO, MT, NC, NE, OK, PA, SC, SD, TN, TX, UT, VA, WI, WY. Private share is the share of non-Medicare hospital discharges covered by private insurance. Standard errors are heteroscedasticity robust and clustered at the state-level. Results are weighted by 2014 state population.
