# Supplementary material for: Changes in inpatient payer-mix and hospitalizations following Medicaid expansion: Evidence from all-capture hospital discharge data
Source: PLoS One. 2017 Sep 28;12(9):e0183616. doi: 10.1371/journal.pone.0183616 (PMC5619726; doi:10.1371/journal.pone.0183616)
Supplement: S2 Fig — (PDF) [file pone.0183616.s006.pdf]

S2 Fig. Difference-in-Differences Payer Share Results through Q3 of 2015.

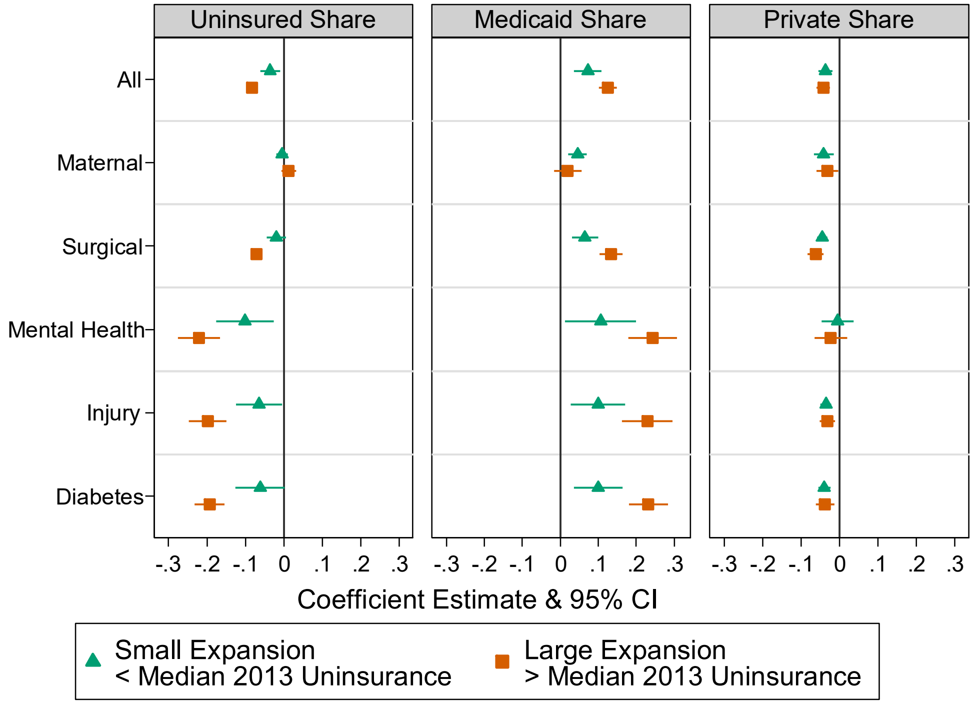

Notes: The figure presents regression-adjusted difference-in-difference estimate and their 95% confidence interval by discharge type. This sample includes the 17 states with data available through the first 3 quarters of 2015. Information on adjusted regression specification may be found in the appendix. Small expansion states include HI, IA, KY, MI, MN, NY large expansion states include CA, CO, NJ, OR, and nonexpansion states include FL, GA, MO, SD, TX, VA, WI. Payer mix is the share of non-Medicare hospital discharges covered by Medicaid, with no source of coverage, and with private insurance coverage. Standard errors are heteroscedasticity robust and clustered at the state-level. Results are weighted by 2014 state population.
