# Supplementary material for: Changes in inpatient payer-mix and hospitalizations following Medicaid expansion: Evidence from all-capture hospital discharge data
Source: PLoS One. 2017 Sep 28;12(9):e0183616. doi: 10.1371/journal.pone.0183616 (PMC5619726; doi:10.1371/journal.pone.0183616)
Supplement: S3 Fig — (PDF) [file pone.0183616.s007.pdf]

S3 Fig. Difference-in-Difference Estimates of Effect of Medicaid Expansion on Payer mix with Controls.

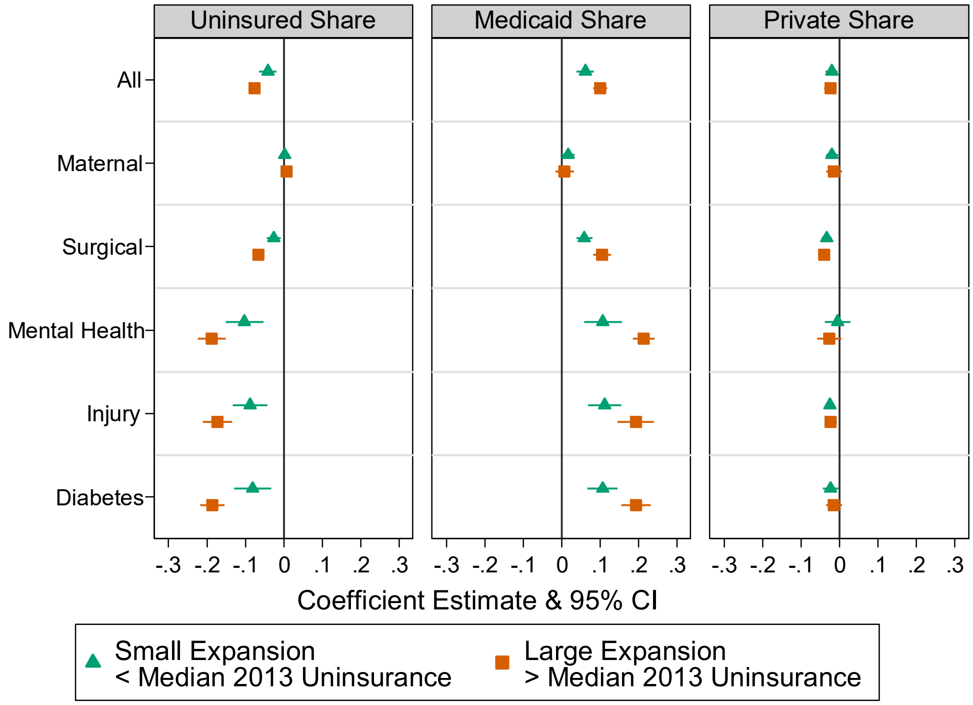

Notes: This figure replicates the results of Figure 1 and S4 Fig with the addition of age, sex, marital status, income and education distributions of the state as well as the unemployment rate as control variables.
