# Supplementary material for: Changes in inpatient payer-mix and hospitalizations following Medicaid expansion: Evidence from all-capture hospital discharge data
Source: PLoS One. 2017 Sep 28;12(9):e0183616. doi: 10.1371/journal.pone.0183616 (PMC5619726; doi:10.1371/journal.pone.0183616)
Supplement: S4 Fig — (PDF) [file pone.0183616.s008.pdf]

S4 Fig. Payer Mix Pre-Trends, No controls.

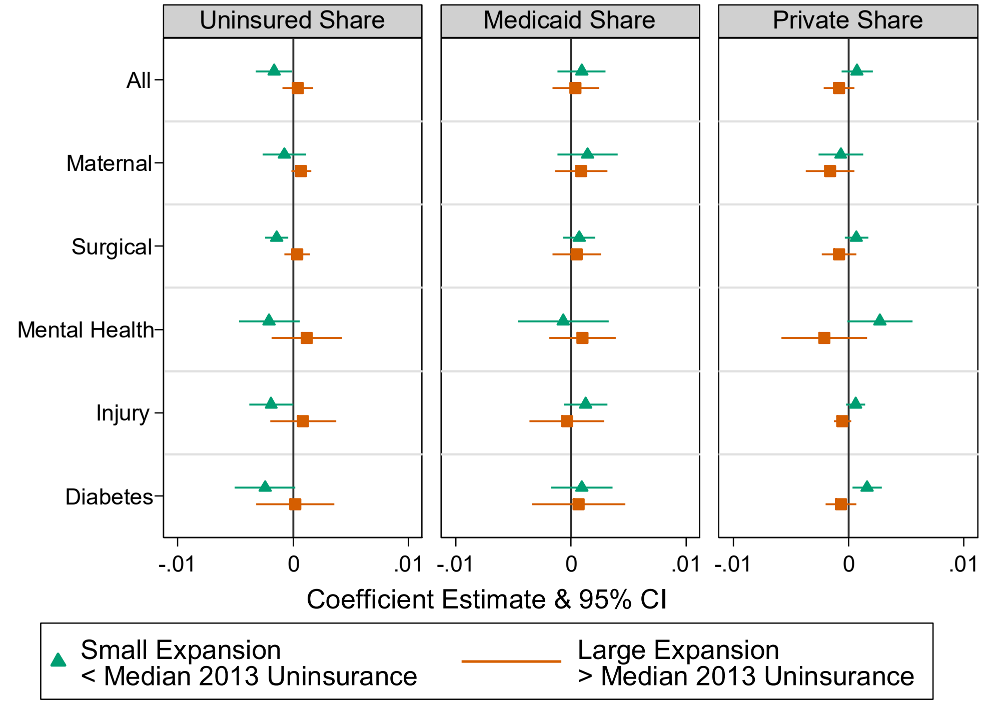

Notes: The figure presents regressions of our payer mix dependent variables on state fixed effects, a linear quarterly trend and an interaction between expansion status and a linear quarterly trend during the period prior to the Medicaid expansion. Small expansion states include HI, IA, IL, KY, MA, MD, MI, MN, NY, RI, VT, WA, WV, large expansion states include AR, AZ, CA, CO, ND, NJ, NM, NV, OR, and non-expansion states include FL, GA, IN, KS, LA, ME, MO, MT, NC, NE, OK, PA, SC, SD, TN, TX, UT, VA, WI, WY. Payer mix is the share of non-Medicare hospital discharges covered by Medicaid, with no source of coverage, and with private insurance coverage. Standard errors are heteroscedasticity robust and clustered at the state-level. Results are weighted by 2014 state population.
