# Supplementary material for: Changes in inpatient payer-mix and hospitalizations following Medicaid expansion: Evidence from all-capture hospital discharge data
Source: PLoS One. 2017 Sep 28;12(9):e0183616. doi: 10.1371/journal.pone.0183616 (PMC5619726; doi:10.1371/journal.pone.0183616)
Supplement: S5 Fig — (PDF) [file pone.0183616.s009.pdf]

S5 Fig. Payer Mix Pre-Trends, With controls.

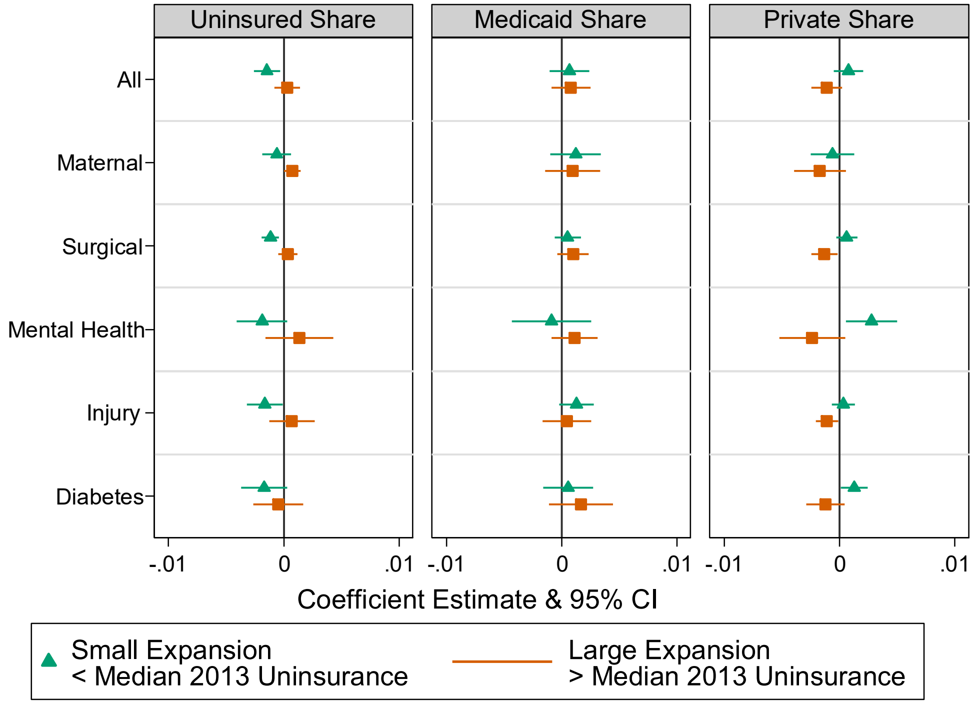

Notes: This figure replicates the results of Appendix 6a with the addition of age, sex, marital status, income and education distributions of the state as well as the unemployment rate as control variables.
