# Supplementary material for: Changes in inpatient payer-mix and hospitalizations following Medicaid expansion: Evidence from all-capture hospital discharge data
Source: PLoS One. 2017 Sep 28;12(9):e0183616. doi: 10.1371/journal.pone.0183616 (PMC5619726; doi:10.1371/journal.pone.0183616)
Supplement: S7 Fig — (PDF) [file pone.0183616.s011.pdf]

S7 Fig. Additional Synthetic Control Time Series.

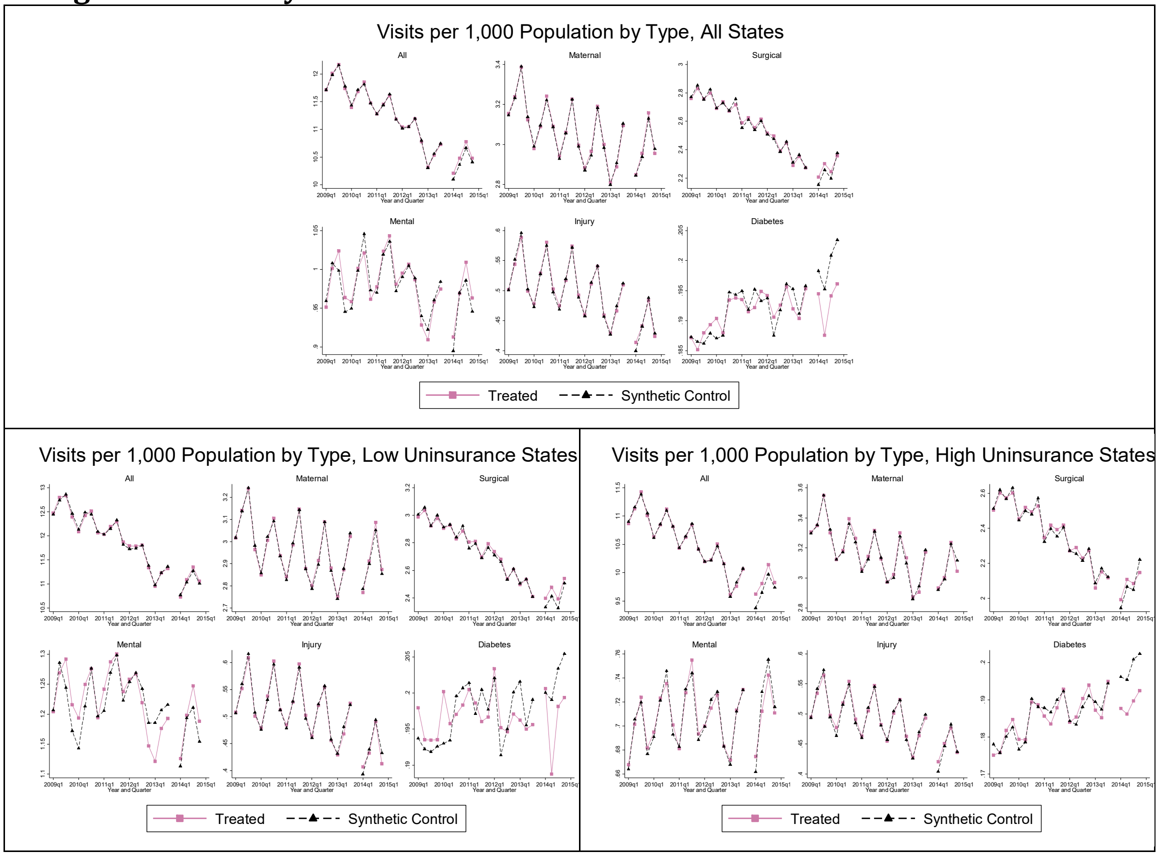

Notes: The figure presents mean time trends for expansion states, small expansion states, and large expansion states weighted by state population in 2014 (treated) and a weighted average of non-expansion states (synthetic controls). The method of choosing weights for the control states are found in the appendix. Outcomes are based on the number of non-Medicare hospital discharges within each type of visit.
