# Supplementary material for: Changes in inpatient payer-mix and hospitalizations following Medicaid expansion: Evidence from all-capture hospital discharge data
Source: PLoS One. 2017 Sep 28;12(9):e0183616. doi: 10.1371/journal.pone.0183616 (PMC5619726; doi:10.1371/journal.pone.0183616)
Supplement: S1 Appendix — (PDF) [file pone.0183616.s012.pdf]

S1 Appendix: Empirical Methods

Payer mix dependent variables were analyzed with a regression based difference-in-differences model. We estimated these regressions using ordinary least squares with heteroscedasticity-robust, state-clustered standard errors using the following regression equation:

$$(1)\ Y_{sq} = \beta_0 + \beta_1 POST \times EXPANSION_{sq} + f_s + g_q + u_{sq}$$

Y is the share of non-Medicare visits covered by Medicaid, without insurance coverage, or private insurance. Post is an indicator variable set to one for visits occurring on or after the 1st quarter of 2014, with all other time periods set to zero. Expansion is an indicator variable set to one for states that expanded Medicaid in 2014 (AR, AZ, CA, CO, HI, IA, IL, KY, MA, MD, MI, MN, ND, NJ, NM, NV, NY, OR, RI, VT, WA, WV). All other states in the dataset (FL, GA, IN, KS, LA, ME, MO, MT, NC, NE, OK, PA, SC, SD, TN, TX, UT, VA, WI, WY) were set to zero. The regression also controls for state fixed-effects (f_s) and quarter-year fixed-effects (g_q). The former controls for time-invariant characteristics between states, and the latter flexibly controls for common changes in visits occurring in both expansion and non-expansion states over time. With these fixed effects included, the interaction between the Post and Expansion indicators gives the difference in the change in visits over time between expansion and non-expansion states, or the difference-in-difference effect.

As discussed below we also explored the robustness of these results to controlling for the age, sex, marital status, income and education distributions of the state as well as the unemployment rate. All time-varying controls are created using the monthly Current Population Survey analyzed at the quarterly level.

A necessary assumption of difference-in-differences is that the dependent variables were trending similarly between Medicaid expansion and non-expansion states before 2014. We tested this assumption by regressing each dependent variable on state fixed effects, a linear quarterly trend and an interaction between expansion status and a linear quarterly trend during the period prior to the Medicaid expansion, and we present these results in S4 Fig. We find that none of these pre-trends are statistically different from zero for payer mix dependent variables, except a small differential trend for the uninsured share of all discharges in small expansion states (-0.00166, 95% CI -0.00325 - -8.01x10^5) and surgical discharges in small expansion states (-0.00142 95% CI -0.00243 - -0.000416). Note that these coefficient sizes are roughly 20 to 200 times smaller relative to the effect size observed after 2014 in Fig 1. Our main specification follows this difference in differences model. In S5 Fig we show pre-trends results from an alternate model where we add demographic control variables. We find there that more of the pre-trends are statistically different form zero.

We applied the same pretrends test to the number of visits per 1,000 population dependent variables, with results presented in S6 Fig. Here we find statistically significant pretends for mental health visits (All: -0.00647 95% CI -0.0104 - -0.00258, Small Expansion: -0.00854 95% CI -0.0135 - -0.00353, Large Expansion: -0.00415 95% CI -0.00681 - -0.00149). Additionally, the magnitudes are generally 5 to 30 times smaller than the main results, which are much larger in relative magnitude than the insurance share pre-trend estimates. Therefore,

number of visits per 1,000 population dependent variables were analyzed with a synthetic control method rather than a difference-in-differences method. Synthetic control chooses a set of weights that, when used to construct a weighted average of data among control states, minimizes the difference in the pre-period values between treatment and control units (Abadie, Diamond, and Hainmueller 2010). The logic is that the weighted average of states that represents the closest match to the pre-treatment trends is the best counterfactual for what would have happened to outcomes post policy in the treatment states, in the absence of the treatment. We first aggregate the treated states into a single treatment unit by taking means of each dependent variable among expansion states, weighted by 2014 state population. We then use Stata's "synth" routine to estimate a set of weights that when used to aggregate the non-treated states into a single "synthetic" control unit match all pre-period quarters of the outcome variable as closely as possible. We construct weights separately for each dependent variable that we analyze. As shown in Fig 2 of the manuscript, after applying these weights to control states, there is very little difference in the time series of each outcome prior to Medicaid expansion between treatment and control states. With this method, the estimated treatment effect is simply the difference between the average over the four post-expansion quarters of the dependent variable in the treatment unit and the synthetic control unit.

We use a Fisher style permutation tests proposed by Kaestner et al. (2015) and Hu et al. (2016) to estimate p-values of these estimates. For each dependent variable, we repeat the synthetic control estimation 1,000 times, but instead of using actual expanding states to construct the treatment unit, we select states randomly to comprise the treatment unit. Unselected states are then used to construct a synthetic control group for this randomly selected treatment unit. For each of these 1,000 simulation, we calculate the root mean squared error between the treatment and synthetic control unit during the post-expansion period and during the pre-expansion period. The ratio of these two values measures how different the two groups are in the post period relative to the pre-period. The proportion of these 1,000 simulations for which this ratio is larger than the ratio in our actual estimation is used as our p-value. In other words, we ask what proportion of these simulations has a wider gap between treatment and control units in the post period, relative to the pre-period, than our actual estimation.

All analysis was performed using Stata.
